# The 25 kDa H_CN_ Domain of Clostridial Neurotoxins Is Indispensable for Their Neurotoxicity

**DOI:** 10.3390/toxins12120743

**Published:** 2020-11-26

**Authors:** Julian Deppe, Jasmin Weisemann, Stefan Mahrhold, Andreas Rummel

**Affiliations:** Institut für Toxikologie, Medizinische Hochschule Hannover, 30625 Hannover, Germany; deppe.julian@mh-hannover.de (J.D.); weisemann.jasmin@mh-hannover.de (J.W.); mahrhold.stefan@mh-hannover.de (S.M.)

**Keywords:** botulinum neurotoxin, BoNT/A, BoNT/B, tetanus neurotoxin, H_CN_ domain, synaptic vesicle protein, gangliosides

## Abstract

The extraordinarily potent clostridial neurotoxins (CNTs) comprise tetanus neurotoxin (TeNT) and the seven established botulinum neurotoxin serotypes (BoNT/A-G). They are composed of four structurally independent domains: the roles of the catalytically active light chain, the translocation domain H_N_, and the C-terminal receptor binding domain H_CC_ are largely resolved, but that of the H_CN_ domain sandwiched between H_N_ and H_CC_ has remained unclear. Here, mutants of BoNT/A, BoNT/B, and TeNT were generated by deleting their H_CN_ domains or swapping H_CN_ domains between each other. Both deletion and replacement of TeNT H_CN_ domain by H_CN_A and H_CN_B reduced the biological activity similarly, by ~95%, whereas BoNT/A and B deletion mutants displayed >500-fold reduced activity in the mouse phrenic nerve hemidiaphragm assay. Swapping H_CN_ domains between BoNT/A and B hardly impaired their biological activity, but substitution with H_CN_T did. Binding assays revealed that in the absence of H_CN_, not all receptor binding sites are equally well accessible. In conclusion, the presence of H_CN_ is vital for CNTs to exert their neurotoxicity. Although structurally similar, the H_CN_ domain of TeNT cannot equally substitute those of BoNT and vice versa, leaving the possibility that H_CN_T plays a different role in the intoxication mechanism of TeNT.

## 1. Introduction

The family of clostridial neurotoxins (CNTs) consists of tetanus neurotoxin (TeNT), the seven established botulinum neurotoxin serotypes (BoNT/A-G), and the recently identified novel types BoNT/HA (aka H & FA), BoNT/X, and eBoNT/J (aka BoNT/En) from *Enterococcus* [1,2,3,4]. Lately, the insecticidal neurotoxin PMP1 isolated from a *Paraclostridium bifermentans* strain has also been identified as a structural homologue of CNTs [5]. Historically, botulinum neurotoxins (BoNTs) are produced by the bacteria *Clostridium botulinum* and are the most poisonous protein toxins known [6]. They cause the disease botulism by blocking release of acetylcholine at the neuromuscular junction, resulting in muscle relaxation. Therefore, BoNTs are extensively used in medical and cosmetic applications [7]. TeNT is similarly toxic, but blocks the release of glycine and GABA at inhibitory neurons, resulting in spastic paralysis and the disease tetanus.

**Figure 1:**
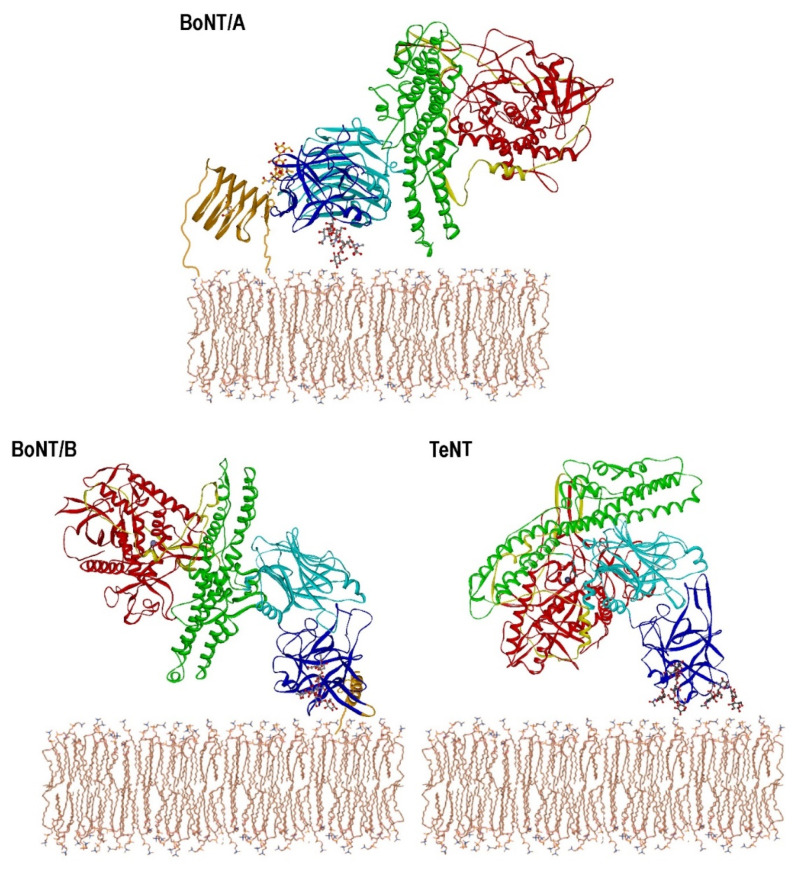
Membrane approach models of crystal structures of BoNT/A, BoNT/B, and TeNT in complex with the luminal parts of their neuronal receptor molecules glycosylated SV2C, Syt-II (orange ribbons) and complex polysialo-gangliosides (balls and sticks). Red ribbons, 50 kDa light chain (LC); yellow ribbons, belt wrapping around the LC; green ribbons, translocation domain (H_N_); light blue ribbon, N-terminal half of H_C_ fragment (H_CN_); dark blue ribbon, C-terminal half of H_C_ fragment (H_CC_).

CNTs are AB-protein toxins of 150 kDa molecular weight (MW), which comprise a 50 kDa light chain (LC) linked by a disulfide bridge to the heavy chain (HC), the latter being divided into three independent domains: H_N_ (50 kDa N-terminal half of HC), H_CN_, and H_CC_ (25 kDa each) (Figure 1) [8,9,10,11]. H_CN_ and H_CC_ together form the 50 kDa H_C_ fragment, which is a well expressed, soluble, non-toxic protein used for example as a vaccine [12]. LC, H_N_, and H_C_ perform individual tasks in the intoxication process: The N-terminal LC is a Zn^2+^-dependent metalloprotease, H_N_ is the translocation domain, and the C-terminal H_C_ fragment is the receptor binding domain [13]. Extensive analyses of the interaction between neuronal receptors and CNT has revealed so far that, with one exception, all receptor binding sites are located within the C-terminal 25 kDa H_CC_ domain of H_C_ [14]. Only the H_CN_ of BoNT/A harbors part of the N-glycan binding site of its protein receptor SV2 [15,16]. Accordingly, a mutant form of recombinant BoNT/A lacking H_CN_ (rAΔH_CN_) showed a 33,000-fold reduced lethality compared to wild-type BoNT/A, presumably due to impaired SV2C binding [17]. However, any additional function of H_CN_A and the role of the 25 kDa H_CN_ domain in all other serotypes still remains elusive.

The BoNT molecular mechanism of action at the cholinergic neuron starts with a low affinity binding of the H_CC_ domain to complex polysialo-gangliosides and subsequent high affinity binding to different synaptic vesicle (SV) protein receptors [14]. In variance, TeNT interacts with extracellular matrix proteins nidogen-1 and 2 first [18], although it remains unclear how this interaction triggers membrane approach of TeNT. In proximity of the plasma membrane TeNT binds to one or two molecules of complex polysialo-gangliosides, a second ganglioside would displace nidogen from its sialic acid binding site. Unlike BoNTs, TeNT is then sorted into vesicles of neutral pH lumen and undergoes retrograde axonal transport to reach the inhibitory neuron. BoNTs undergo receptor-mediated endocytosis via synaptic vesicles. Upon acidification of lumen, the BoNTs change their conformation and presumably insert a pore or channel into the vesicle membrane [19]. The partially unfolded LC is translocated through the pore formed by the H_N_ domain into the neuronal cytosol and is released after reduction of the disulfide bridge connecting LC and HC by thioredoxin and its reductase [19,20,21]. Here, the hydrolysis of a specific soluble *N*-ethylmaleimide-sensitive factor attachment protein receptor (SNARE) protein takes place; in the case of BoNT/A synaptosome-associated protein of 25 kDa (SNAP-25) is specifically cleaved at Q197-R198 [22,23], while BoNT/B and TeNT cleave the identical peptide bond Q76-F77 in synaptobrevin/VAMP-2 [24]. This cleavage results in inhibition of the neurotransmitter release into the synaptic cleft, leading to flaccid and in the case of TeNT to spastic paralysis, respectively, and eventually to death by respiratory failure.

BoNT/A, B, E, F, and G and TeNT all possess a single ganglioside binding site (GBS) comprising a conserved motif [25,26,27], which specifically recognizes complex polysialo-gangliosides albeit with submicromolar affinity [28,29,30,31]. In addition to this conserved GBS, TeNT comprises a second ganglioside binding site interacting with sialic acids as well as defined peptide motifs found, e.g., in nidogen-1 and 2 [18,32,33,34]. The SV proteins synaptotagmin-I and -II (Syt-I/-II) serve as protein receptors for BoNT/B, G, and the mosaic type BoNT/DC [35,36,37,38,39,40,41], whereas the three isoforms of the synaptic vesicle glycoprotein 2 (SV2A-C) were identified as protein receptors for BoNT/A [42,43]. Furthermore, glycosylated SV2A and B exclusively serve as protein receptors for BoNT/E [44]. Only the luminal domain 4 (LD4) connecting TMD 7 and 8 of all three SV2 isoforms interacts with BoNT/A H_C_ [42,43] and folds into a right-handed, quadrilateral β-helix (Figure 1), which binds to BoNT/A next to the interface of H_CN_ and H_CC_ [15,45,46]. An N-glycan conserved in SV2A-C increases the affinity of BoNT/A to SV2C 12-fold by binding with its glycan core into a cleft between the H_CN_ and H_CC_ domains [16,47]. This SV2C-binding site locates almost opposite to the GBS in H_CC_A with a distance of approximately 40 Å. In contrast, the Syt binding site locates at the tip of the BoNT/B, G, and DC H_CC_ domains in close proximity to the single GBS [27,30,46,48,49].

The isolated 25 kDa domains H_CN_ and H_CC_ of BoNT/A, B, and G are readily produced, but only H_CC_ of BoNT/B and G display binding to their protein receptors Syt-I/-II [39]. A fusion protein of enhanced green fluorescent protein (EGFP) and BoNT/A H_CN_ (EGFP-H_CN_/A) binds to the plasma membrane of the neuronally differentiated NSC-34 cells and to spinal cord motoneurons all along the cell surface in discrete spots identified as sphingomyelin-enriched membrane microdomains, but is not internalized [50]. Interestingly, EGFP-H_CN_/A binds with a similar pattern also to the plasma membrane of the epithelial cell lines HeLa and Caco-2, but does not transcytose polarized T84 monolayers as H_C_A does [51]. However, the epithelial binding could not be demonstrated for the entire receptor binding domain H_C_A comprising H_CN_ and H_CC_ (“EGFP and EGFP-H_C_/A did not bind to HeLa cells.”; L. Muraro PhD thesis, 2009, Figure 20, p. 56). In a dot-blot assay, EGFP-H_CN_/A binds phosphatidylinositol phosphates (PI(5)P > PI(3)P > PI(4)P) [50].

**Figure 2:**
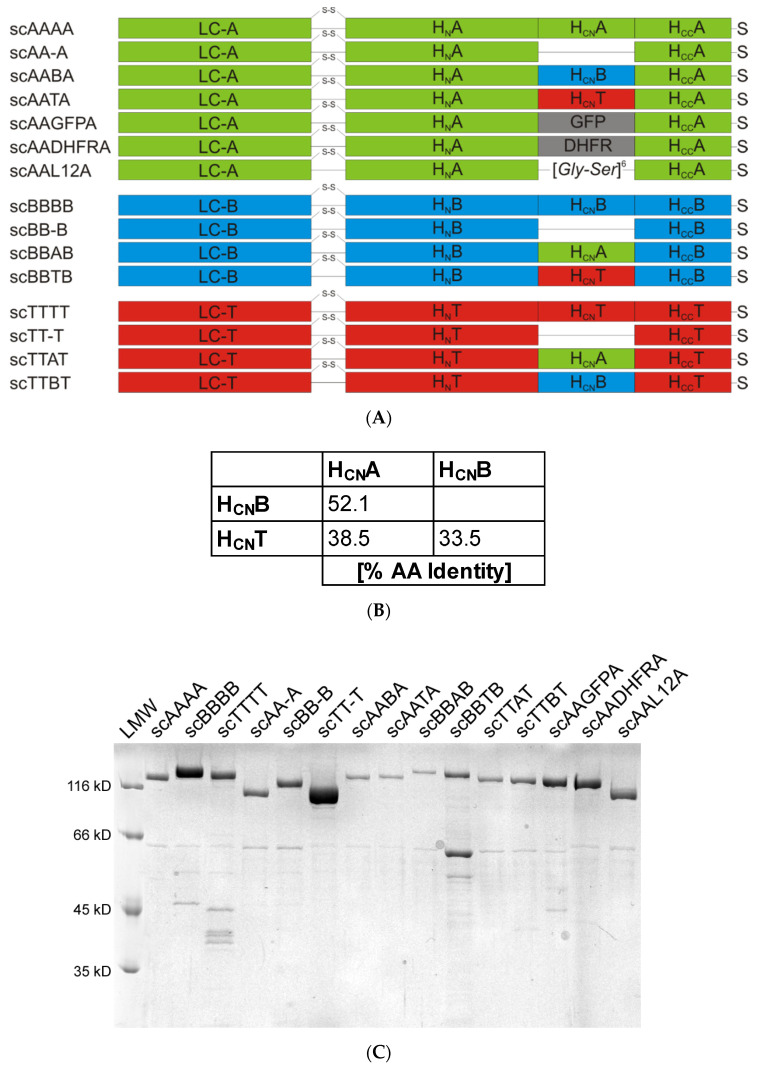

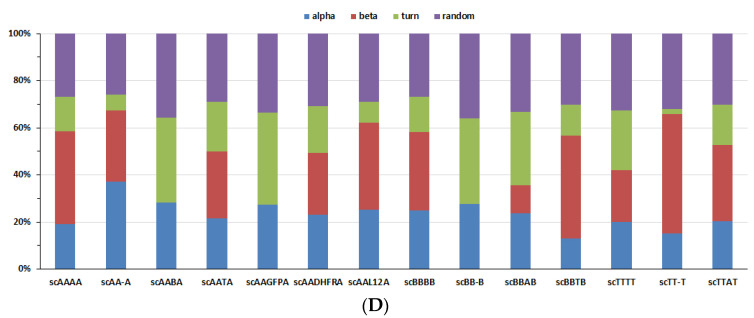
(**A**) Schematic overview of the generated BoNT/A, B, and TeNT deletion mutants and hybrids. scAA-A lack residues K871-N1093, scBB-B S858-Y1080, and scTT-T I880-I1111, respectively. GFP, green fluorescent protein; DHFR, dihydrofolate reductase; L12 = [GlySer]_6_-linker; sc single chain. A capital letter according to the serotype origin was assigned to each of the four domains (LC, H_N_, H_CN_, H_CC_). (**B**) AA sequence identity of the H_CN_ domains of BoNT/A, BoNT/B, and TeNT. (**C**) Reducing 10% SDS-PAGE analysis of the 12 novel deletion mutants and chimeras (detection by Coomassie stain). (**D**) Secondary structure estimation of all mutants by CD-spectroscopy. Proportion of alpha-helices, beta-sheet, turn, and random coil is given in %.

In this study, we deleted or substituted the H_CN_ domain within BoNT/A, BoNT/B, and TeNT holotoxins to obtain insight into its function. Corresponding crystal structures and biochemical data guided us to define the sequence comprising the H_CN_ domains (Figure 1 and Figure 2). At first, mutants of BoNT/A, BoNT/B, and TeNT were generated either lacking their H_CN_ domains or containing H_CN_ domains from one of the other two CNTs. Their residual biological activity was tested using the mouse phrenic nerve hemidiaphragm (MPN) assay. Pull-down assays with GST-fusion proteins of their SV protein receptors as well as with ganglioside incorporated liposomes verified the functionality of the binding areas and thereby correct folding of the H_CC_ domains. Secondary structure analyses by circular dichroism spectroscopy (CD) of mutants confirmed these findings.

A main function of the H_CN_ domain besides contributing to N-glycan binding of SV2 in BoNT/A might be the correct positioning of the H_CC_ domain relative to the remaining LH_N_ of BoNT to provide access of the synaptic protein receptor to its binding pocket as well as to facilitate a subsequent pH-dependent insertion of H_N_ into the vesicle membrane. Proteins of similar size but different folding, which could act as a rigid spacer, cannot adopt that role. In summary, these data progress the understanding of the H_CN_ domain and its role in the mechanism of action of CNTs.

## 2. Results

### 2.1. Generation of H_CN_ Deletion Mutants and Hybrids of BoNT/A, B and TeNT

Crystal structures of BoNT/A, BoNT/B and TeNT holotoxins, primary sequence alignments, and biochemical data were used to define the sequences comprising the H_CN_ domains (Figure 1 and Figure 2A). The H_CN_A is defined from K871-N1093, H_CN_B covers S858-Y1080, and H_CN_T spans from I880-I1111. At first, mutants of BoNT/A, BoNT/B, and TeNT were constructed lacking their H_CN_ domains, yielding scAA-A ΔK871-N1093, scBB-B ΔS858-Y1080, and scTT-T ΔI880-I1111.

Due to the high amino acid (AA) sequence identity of 52% between H_CN_A and H_CN_B as well as to H_CN_T (33% and 35%, respectively, Figure 2B) and a highly similar fold as reflected by DALI Z-scores > 39.5, we attempted to swap H_CN_ domains between the three neurotoxins yielding six full-length hybrid toxins (Figure 2A).

Additionally, the H_CN_ domain of BoNT/A was substituted with non-clostridial polypeptides of similar size but different conformation like the 26 kDa green fluorescent protein (GFP), the 21 kDa dihydrofolate reductase (DHFR), or a 12 amino acid long peptidic GlySer-linker (L12).

The three deletion mutants lacking H_CN_ (AA-A, BB-B, TT-T; a capital letter according to the serotype origin was assigned to each of the four domains LC, H_N_, H_CN_, and H_CC_) and all full-length hybrids were recombinantly expressed as single-chain (sc) proteins in *Escherichia coli*. Changes in molecular weight due to domain deletion (e.g., scAA-A, 124 kDa; scBB-B, 127 kDa; scTT-T, 126 kDa) or exchanged domains are clearly visible by SDS-PAGE analysis (Figure 2C). All deletion mutants and hybrids were isolated as soluble proteins in good purity by a single step affinity chromatography. Protein yields ranged from 0.02–5 mg/L culture volume. Highest yield was obtained for the wild-type proteins scTTTT with 5 mg/L followed by scBBBB (1.5 mg/L) and scAAAA (0.5 mg/L). Removal of H_CN_ domain dropped yield for scBB-B to 0.02 mg/L and for scTT-T to 0.5 mg/L, but increased yield of scAA-A to 1 mg/L. Apart from scBBTB, all other full-length mutants were isolated with yields between 0.15–2.0 mg/L. This indicates that the domain exchange in the majority of mutants did not impair the tertiary structure and solubility of the CNTs.

However, protein yield is only an indirect measure for correct protein folding. We therefore conducted secondary structural analysis of the mutants using CD spectroscopy, since deletion of the whole H_CN_ domain, its replacement either by another H_CN_ domain, or an even non-CNT domain like GFP or DHFR generates a completely novel protein with novel characteristics like individual melting temperature. The alpha-helical content of the three wild-type CNTs (scAAAA, scBBBB, and scTTTT) ranges between 19–24% (Figure 2D), which is in good accordance with their full-length crystal structures (Figure 1) [8,9,10]. Alpha-helices are almost exclusively found in LC and H_N_, but hardly in H_C_. Therefore, the absolute alpha-helical content should not alter in the deletion mutants and in the CNT hybrids, but the relative alpha-helical content will increase due to the reduction of molecular weight by 25 kDa. Indeed, the hybrids display an alpha-helical content from 13–28%, which is comparable to their wild-type counterparts. As expected, scAA-A and scBB-B show an increased proportion of 37% and 28% compared to 19% and 25% of scAAAA and scBBBB, respectively. Beta-sheet content and turns are rather variable over all constructs, but the random coil, a clear indicator of unfolded protein, is very narrow between 26–36%. Since the purity of some constructs like scBBTB is below 90%, one also has to consider the impurities’ effect on the outcome. Altogether, secondary structural analysis of the mutants using CD spectroscopy revealed no unfolded protein or even major changes in secondary structure. Minor changes cannot be excluded and will be only exhibited by X-ray crystallography, but are inherent when generating novel molecules. Functional analysis described below will shed further light on the structural integrity of the mutants.

### 2.2. Determination of Residual Biological Activity Using the Mouse Phrenic Nerve Hemidiaphragm (MPN) Assay

**Figure 3:**
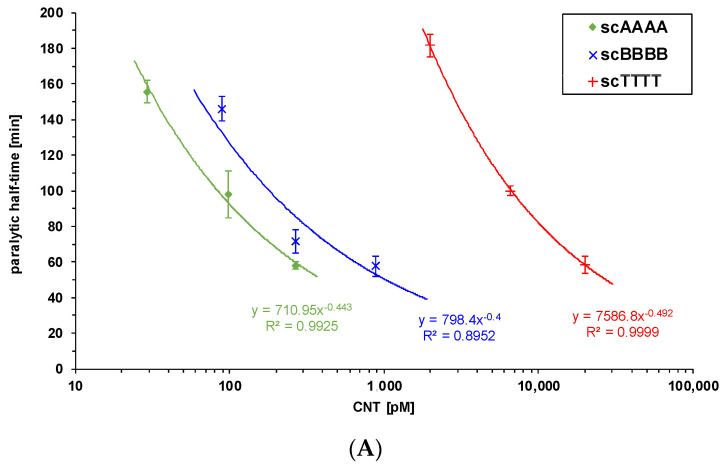

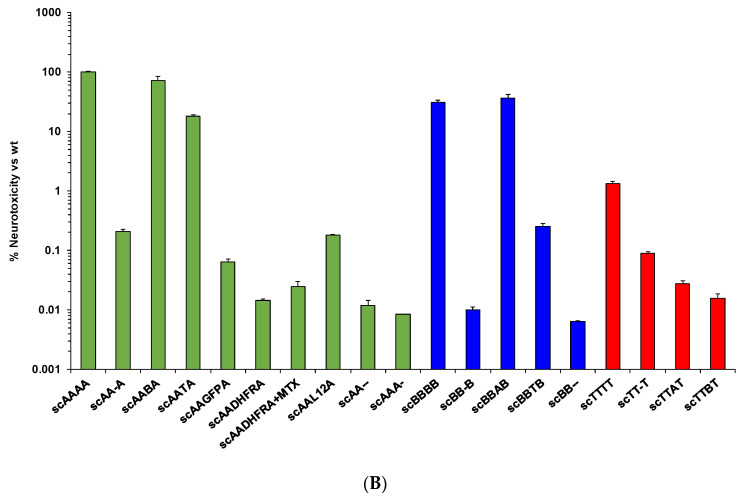
Determination of biological activity of mutants by the MPN assay. CNTs were added to electrically stimulated mice hemidiaphragm preparations, isometric contractions were recorded, and the time required to decrease the amplitude to 50% of the starting value was determined. (**A**) Dose-response curves of wild-type constructs scAAAA, scBBBB, and scTTTT with their corresponding fitted power functions (n = 3–4 biol. replicates; mean ± SD). (**B**) Neurotoxicity of mutants. All measurements (n = 3–4 biol. replicates; mean ± SD) are referenced to scAAAA dose response power function; MTX, 0.6 µM methotrexate (induces rigid structure of DHFR).

The residual biological activity of all mutants was tested using the mouse phrenic nerve hemidiaphragm (MPN) assay, which constitutes an excellent system to determine the pharmacodynamic properties without performing an animal experiment [52]. First, individual dose-response curves were generated for the three wild-type proteins scAAAA, scBBBB, and scTTTT, to which power functions for calculating the relative toxicity of mutants were fitted. scAAAA displays the highest potency, while scBBBB is about 2.3-fold less potent than scAAAA (Figure 3A confirming previous results [53]). scTTTT is the least potent neurotoxin in the MPN assay (32-fold less potent than scAAAA) [54], because the motoneuron of the hemidiaphragm preparation constitutes TeNT’s physiological uptake site into vesicles with pH-neutral lumen, but its main site of action is the inhibitory interneuron absent in the MPN assay. At higher concentrations, a small fraction of TeNT seems to be taken up into acidifying vesicles, thereby releasing TeNT LC into the cytosol of the motoneuron and blocking acetylcholine release like BoNT.

Deletion of the H_CN_ domain in TeNT reduced the biological activity of scTT-T in the hemidiaphragm tissue about 13-fold compared to its wild-type scTTTT, whereas scAA-A and scBB‑B deletion mutants displayed 480-fold and 4000-fold decreased activity compared to their respective wild-type forms. Using scAAAA as common reference, scTT-T maintains nine-fold higher potency than scBB-B (0.09 vs. 0.01%) and is only 50% less potent than scAA-A (0.21%; Figure 3B). Replacement of the H_CN_ domain in TeNT by H_CN_A and H_CN_B decreased the potency of scTTAT and scTTBT further to 0.027% and 0.016%, respectively. In contrast, swapping H_CN_ domains between BoNT/A and B hardly impaired activity of scAABA (72%), while scBBAB even profits from insertion of H_CN_A (106% of scBBBB potency). The substitution of H_CN_A by H_CN_T was tolerated; scAATA still displayed 18% potency of wild-type, but potency of scBBTB was drastically reduced to 0.25% of scAAAA.

Additionally, the H_CN_ domain of BoNT/A was substituted with non-clostridial polypeptides of similar size like the 26 kDa GFP, the 21 kDa DHFR, or the peptidic linker L12. Whereas the scAAL12A construct behaves like the BoNT/A deletion mutant scAA-A (0.18%), the substitution of the H_CN_A domain by the rigid β-barrel structure of GFP decreased activity. Despite DHFR being a flexible protein, it is not well tolerated, as can be seen by the potency of 0.015% of scAADHFRA. Freezing the DHFR conformation by the addition of 0.6 µM methotrexate (MTX), a chemotherapy agent that acts as an immune system suppressant by inhibiting tetrahydrofolate synthesis by DHFR, potency of scAADHFRA increases to 0.025%, which is according to a paired *t*-test of these two experiments non-significant (*P* = 0.183). MTX on its own did not influence neurotransmitter release. Deleting the entire 50 kDa H_C_A domain (scAA--) yields a nearly inactive molecule (0.012% potency; Figure 3B), as demonstrated earlier [55].

### 2.3. Pull-Down Assays Employing GST-rSV2C 454-579, GST-rSyt-II 1-61 and ghSV2CLD-Fc

**Figure 4:**
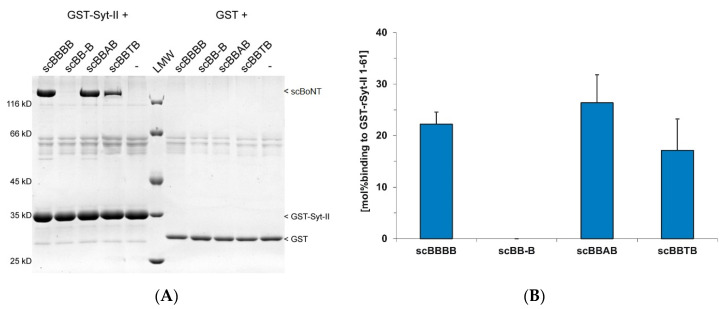
GST-pull-down assay of BoNT/B mutants employing GST-Syt-II 1-61. (**A**) GST-Syt-II 1-61 fusion-protein, protein receptor of BoNT/B was immobilized on glutathione-sepharose beads and subsequently incubated with the indicated wild-type/mutants (250 nM) for 2 h at 4 °C. After washing, proteins bound to the solid phase were analyzed by SDS-PAGE and visualized by Coomassie staining. Parallel experiments with GST served as negative controls. (**B**) Quantitative analysis of wild-type/mutants bound to GST-Syt-II 1–61 (n = 3 biol. replicates; mean ± SD). The value “mol% binding” is the ratio of band intensity of the respective GST fusion protein as immobilized bait and the bound BoNT as ligand/prey corrected by the molecular weight ratio of BoNT vs. GST fusion protein. Unspecific ligand binding to GST was subtracted.

The previous demonstration that isolated H_CC_B interacts with its protein receptors Syt-I/-II [39] as well as the identification of the Syt-II binding site in BoNT/B at the tip of the H_CC_ domain [27,48,49] sets the ideal tool to confirm the correct folding of the H_CC_ domain within the deletion mutant scBB‑B and the corresponding BoNT/B hybrids as well as to verify the integrity of the receptor binding site. Surprisingly, the deletion mutant scBB-B lacks any interaction with its protein receptor Syt-II (Figure 4). In contrast, the hybrids BBAB and BBTB display a binding of 26.4 mol% and 17.2 mol% to GST-Syt-II 1-61, respectively, compared to 22.2 mol% of BoNT/B wild-type, indicating an intact H_CC_B and a fully functional Syt-binding site.

**Figure 5:**
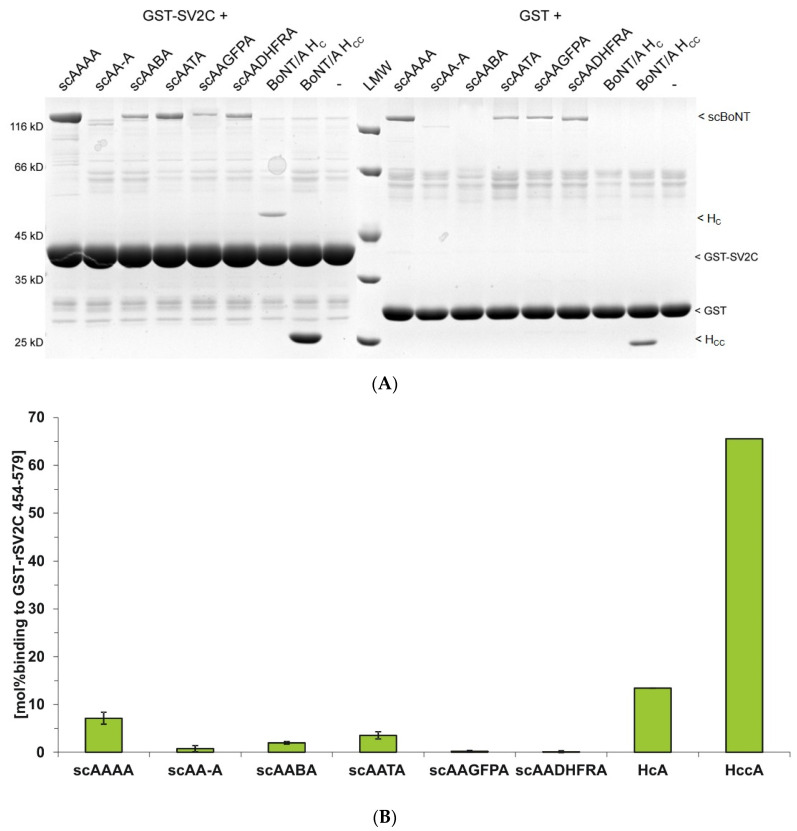
GST-pull-down assays of BoNT/A mutants employing GST-rSV2C 454–579. (**A**) GST-rSV2C 454–579 fusion-protein, protein receptor of BoNT/A (Luminal domain 4 of rat synaptic vesicle protein 2C), was immobilized on glutathione-sepharose beads and incubated with indicated wild-type/mutants (250 nM) for 2 h at 4 °C. Proteins bound to the solid phase were analyzed by SDS-PAGE and visualized by Coomassie staining. Parallel experiments with GST only served as negative controls. (**B**) Quantitative analysis of wild-type/mutants bound to GST-rSV2C 454–579 (n = 3 biol. replicates; mean ± SD). The value “mol% binding” is the ratio of band intensity of the respective GST fusion protein as immobilized bait and the bound BoNT as ligand/prey corrected by the molecular weight ratio of BoNT vs. GST fusion protein. Unspecific ligand binding to GST was subtracted.

Analogously, the correct folding of the H_CC_ domain within the deletion mutant scAA-A and the BoNT/A hybrids was verified employing non-glycosylated GST-rSV2C 454–579. Here, the deletion mutant scAA-A still displays interaction with its protein receptor SV2C albeit with lower affinity than BoNT/A wild-type (0.76 mol% vs. 7.1 mol%; Figure 5). Binding of the hybrids scAABA and scAATA to GST-rSV2C 454–579 is moderately impaired (2.0 mol% and 3.5 mol%), indicating an intact folded H_CC_A in both proteins and a largely functional and accessible SV2C-binding site. The hybrids with the non-clostridial H_CN_ substitutions scAAGFPA and scAADHFRA display hardly detectable binding to GST-rSV2C 454–579. As a control, the isolated H_C_A fragment showed 13 mol% binding to GST-rSV2C, twice as much as full-length scAAAA. Binding of the isolated H_CC_A to GST-rSV2C was almost stoichiometric with 65 mol%, which might be explained by the increased rotational freedom, an improved accessibility of SV2C to its binding site (no shielding by LC and H_N_), or unspecific interactions with the exposed hydrophobic H_CN_ interface.

**Figure 6:**
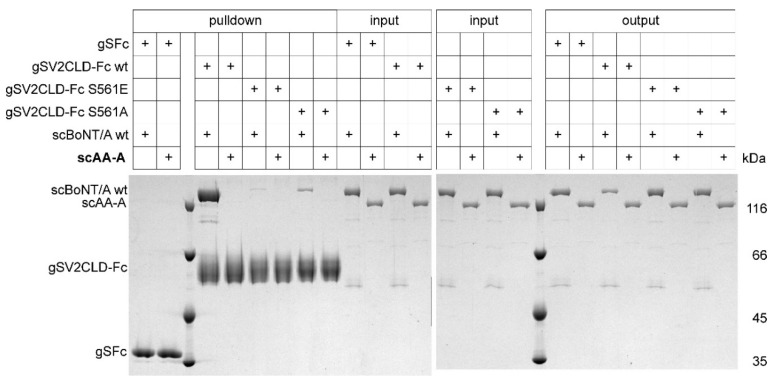
Pull-down assay with glycosylated SV2C and scAA-A and BoNT/A wild-type. 100 pmol of wild-type and mutant glycosylated gSV2CLD-Fc were immobilized to protein G sepharose matrix and incubated with 250 nM (50 pmol, input) of scAA-A or BoNT/A wild-type, respectively, for 2 h at 4 °C. After removal of supernatant (output) and washing of the beads, bound proteins (pulldown) were visualized by SDS-PAGE and Coomassie Blue staining.

Recently, we showed the importance of the complex glycan-branch at N559 of SV2C-LD4, adding a third anchor point beside a ganglioside and the SV2C-LD4 peptide, for BoNT/A neuronal cell surface binding and uptake [47]. Since the N559-glycan was shown to bind predominantly to residues assigned to the H_CN_A domain [16], we deepened our analysis and employed our glycosylated human SV2C-LD4 Fc fusion protein gSV2CLD-Fc in a pull-down assay to investigate the binding of the scAA-A deletion mutant. As seen previously, BoNT/A wild-type showed almost stoichiometric high-affinity binding to gSV2CLD-Fc wild-type (Figure 6). Preventing the glycosylation of N559 by exchanging S561 of the PNG motif N-X-S/T to alanine reduced the binding of BoNT/A wild-type by ~10-fold, reproducing our previous results about the importance of the N559-glycan [47]. Since residue S561 is part of the β-strand mediating the protein–protein interaction with H_CC_A, we mutated S561 also to glutamate to destroy that interaction. Indeed, BoNT/A wild-type hardly bound to gSV2CLD-Fc S561E. In contrast to BoNT/A wild-type, binding of scAA-A to any gSV2CLD-Fc was close to the limit of detection of Coomassie staining. Since scAA-A displays only 10% of BoNT/A wild-type binding to non-glycosylated GST-rSV2C 454–579, it is understandable that such a signal cannot be seen considering the weak signal for BoNT/A wild-type binding to gSV2CLD-Fc S561A. Surprisingly, the presence of the N559-glycan in gSV2CLD-Fc wild-type does not rescue absence of H_CN_A, but rather destabilizes protein–protein interaction with scAA-A (Figure 6). 

### 2.4. Ganglioside Binding of CNT Deletion Mutants and Hybrids in Liposome Binding Assay

To analyze the ability of the deletion mutants and hybrids to bind to gangliosides, isolated polysialo-ganglioside GT1b was incorporated into liposomes to provide a membranous environment.

**Figure 7:**
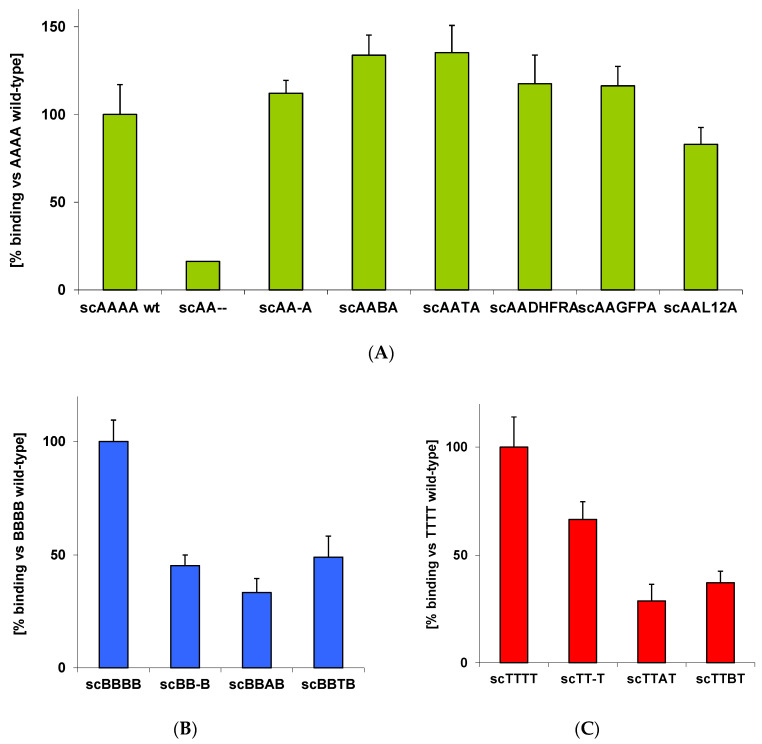
Ganglioside binding of CNT deletion mutants and hybrids in liposome binding assay. An emulsion of GT1b-functionalized RhodamineB-DHPE liposomes was incubated with the respective CNTs for 2 h at RT. Bound CNTs were sedimented, and incorporated fluorophore RhodamineB was liberated upon Triton X-100 lysis and determined by fluorescence microplate reader. (**A**) Ganglioside binding of BoNT/A deletion mutants and hybrids (n = 3 biol. replicates ± SD). (**B**) Ganglioside binding of BoNT/B deletion mutants and hybrids (n = 2 biol. replicates ± SD). (**C**) Ganglioside binding of TeNT deletion mutants and hybrids (scTTTT n = 7, scTT-T n = 5, scTTAT and scTTBT n = 3 biol. replicates; mean ± SD).

All BoNT/A-based hybrids as well as the deletion mutant sAA-A showed ganglioside binding virtually indistinguishable from BoNT/A wild-type (Figure 7). Hence, absence of H_CN_A or its replacement by other domains did not influence functionality of the conserved GBS in H_CC_A. As control, the LH_N_ of BoNT/A, AA--, lacking the complete receptor binding domain H_C_A was analyzed and showed only background binding as expected.

BoNT/B- and TeNT-based hybrids as well as the deletion mutants showed all binding to liposome-incorporated GT1b albeit at clearly reduced levels of 30–65% compared to wild-type BoNT/B and TeNT.

### 2.5. Secondary Structure Analyses of the Deletion Mutant AA-A

**Figure 8:**
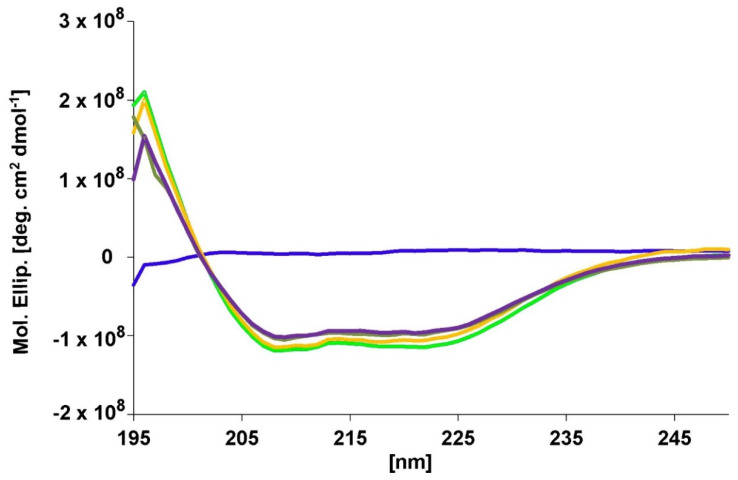
Far-UV spectra of free H_CC_A (blue trace), scAA--(green trace), scAA-A (lime trace), and a stoichiometric mixture of H_CC_A/scAA-- (purple trace) as well as the arithmetical combination of H_CC_A and AA-- (orange trace). Accordingly, the combination of H_CC_A and scAA-- yielded a spectrum similar to that of AA-A.

The determination of secondary structure by CD spectrometry provides a powerful technique to analyze mutants in comparison to their wild-type molecule. To verify whether H_CC_A within the deletion mutant scAA-A adopts the same fold as isolated H_CC_A, which shows high affinity binding towards GST-rSV2C 454–579 (Figure 5), the CD spectrum of scAA-A was determined (Figure 8). Subsequently, AA-- lacking the complete receptor binding domain H_C_A, the isolated H_CC_A, and a stoichiometric mixture of both mimicking AA-A were determined. In accordance with the BoNT/A crystal structure, H_CC_A displays predominantly β-sheet, turns, and random coil and hardly helical parts (Figure 8). Accordingly, its molar ellipticity signal was small. Isolated AA-- displays a high content of α-helices, especially due to the long α-helices in the translocation domain H_N_, which is visible in the minima at 210 and 220 nm of its spectrum (Figure 8). Measurement of AA-A yields a minimally lower molar ellipticity than AA--, which indicates a higher β-sheet content due to the presence of the H_CC_A. Finally, the trace of a stoichiometric mixture of AA-- and HccA nicely overlaps with that of AA-A, demonstrating that isolated AA-- and H_CC_A in total comprise the same secondary structure as AA-A on its own. A theoretical addition of the spectra of H_CC_A and AA-- yielded a spectrum virtually indistinguishable from that of the stoichiometric mixture of AA-- and HccA.

## 3. Discussion

The 150 kDa CNTs are folded into four distinct domains and to three of those, LC, H_N_, and H_CC,_ a role in the mechanism of action at the neuronal site has been ascribed and experimentally proven [13,56,57,58]. This evidence is missing for the 25 kDa H_CN_ domain sandwiched between the translocation domain H_N_ and the H_CC_ domain comprising receptor binding sites.

Although an isolated BoNT/A H_CN_ (EGFP-H_CN_/A) binds to the plasma membrane of neurons in discrete spots identified as sphingomyelin-enriched membrane microdomains without being internalized [50], a directed mutagenesis analysis of a site suggested for interaction with PIP did not confirm this hypothesis [17]. In the latter study, also a BoNT/A H_CN_ deletion mutant was generated, replacing the segment I874-Q1091 by two glycines (=rAΔH_CN_) and subsequently characterized in detail. Its lethality was reduced 33,000-fold as well as its biological activity in primary neuronal cultures, clearly demonstrating the dramatic loss in the overall biological activity upon deleting the H_CN_ from BoNT/A. Their results are in accordance with the 500-fold reduction of biological activity of our deletion mutants scAA-A and scAAL12A (Figure 3B). Their rAΔH_CN_ lacked high-affinity binding to rat cultured cerebellar granule neurons at 4 °C as well as subsequent internalization [17]. It remains unclear whether the rAΔH_CN_ maintained its ability to bind to complex polysialo-gangliosides. Our deletion mutants scAA-A or scAAL12A showed binding to GT1b incorporated in liposomes similar to BoNT/A wild-type (Figure 7A), indicating that the lack of H_CN_A does not interfere with ganglioside binding taking place in H_CC_A. In contrast, the protein–protein interaction of scAA‑A with its non-glycosylated protein receptor SV2C was reduced about 10-fold (Figure 4A), although the SV2C binding site was allocated to a β-strand in H_CC_A [15,45] present in scAA-A. Similar findings were obtained for rAΔH_CN_ [17]. However, the SV2C site is in close proximity to the H_CN_ interface and in the absence of H_CN_A as in scAA-A the translocation domain H_N_ might shield access to the SV2 site in H_CC_A (Figure 1). In contrast, in isolated H_CC_A, the SV2C site is freely accessible, explaining its high affinity binding to GST-rSV2C 454–579 (Figure 4A). This obstructing effect in scAA-A is even further pronounced by testing binding to glycosylated SV2C (Figure 5). The high affinity interaction of BoNT/A wild-type with SV2C based upon the bidentate binding of the N-glycan and two β-sheets of the quadrilateral SV2C helix cannot be established, since the N-glycan binding site mainly located in H_CN_A is absent [16]. On top, the N-glycan’s presence might hinder access of the two β-sheets of SV2C to its site in H_CC_A directly next to H_N_ even more.

We extended our study on the role of H_CN_ beyond the work of Wang et al. [17] by analyzing the effect of deleting H_CN_ also in BoNT/B and TeNT. Interestingly, scBB-B displays a 10-fold lower biological activity than scAA-A, whereas the biological activity of scTT-T at the motoneuron is in the range of that of the scAA-A deletion mutant. Since TeNT wild-type is just 13-fold more potent than scTT-T at the motoneuron, we speculate whether absence of H_CN_T reduces uptake of scTT-T into neutral vesicles and subsequent retrograde axonal transport. Low potency of scBB-B could be caused by folding problems in H_CC_B as illustrated by low protein yields and lack of detectable binding to its protein receptor Syt-II (Figure 4B). However, scBB-B still shows 40% binding towards GT1b-liposomes compared to BoNT/B wild-type and isolated H_CC_B displays solid binding of Syt-I and -II [39]. Since the Syt binding site is located at the tip of H_CC_B [27,48,49], its access is unlikely to be obstructed by H_N_ in scBB-B.

**Figure 9:**
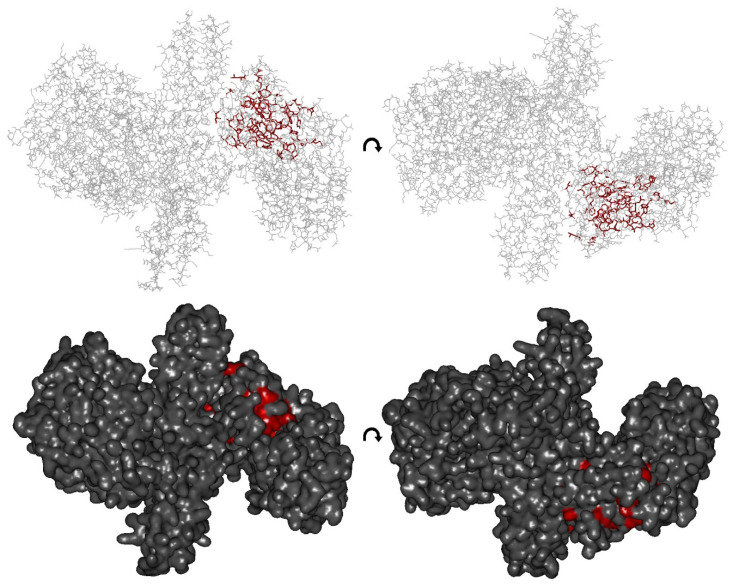
Strictly conserved residues in H_CN_ of BoNT/A, B, and TeNT. Based upon primary sequence alignment of BoNT/A, B, and TeNT, strictly conserved residues are highlighted in red in the BoNT/A H_CN_ structure (3BTA.pdb). Upper panel, stick representation, lower panel surface representation, BoNT/A shown with 180° rotation. The majority of strictly conserved residues are at the hydrophobic core of H_CN_ and dictate its overall fold. Only three strictly conserved residues (I931, N934, L1023, numbering according to BoNT/A) are in close proximity to H_N_ and seem to interact. At the opposite interface, there are four strictly conserved residues (R947, P949, K950, W1013).

Besides deleting H_CN_ in BoNT/A, BoNT/B, and TeNT holotoxins, we also attempted to swap H_CN_ between the CNTs analyzed here. This approach was fueled by our previous studies in which either the whole H_C_ fragment or just the H_CC_ domain of BoNT/A, BoNT/B, and TeNT were successfully replaced with one of the other two types [53,54]. Replacement of the H_CN_ domain in TeNT by H_CN_A and H_CN_B reduced the biological activity similarly to scTT-T by about 95%, indicating that the sheer presence of a lectin-like fold of 25 kDa does not contribute to the biological activity of TeNT (Figure 3B), which is in line with the position of conserved residues in the H_CN_ domain. Based upon the primary sequence alignment of BoNT/A, B, and TeNT, the strictly conserved residues are highlighted in a BoNT/A H_CN_ structure (Figure 9). Here, the majority of strictly conserved residues are at the hydrophobic core of H_CN_ and dictate its overall fold. Only three strictly conserved residues (I931, N934, L1023, numbering according to BoNT/A) are in close proximity to H_N_ and seem to interact. At the opposite interface, four strictly conserved residues (R947, P949, K950, W1013) seem to interact with H_CC_. The majority of other inter-domain interactions are mediated by serotype specific residues explaining the low biological activity of the TeNT hybrids. However, which features in H_CN_T fulfill these specific requirements is unclear.

Swapping H_CN_ domains between BoNT/A and B only moderately impairs biological activity of scAABA and marginally increases biological activity of scBBAB vs. BoNT/B wild-type (Figure 3B). Accordingly, binding of scBBAB to Syt-II is unaltered, indicating a functionally folded HccB, while GT1b binding is approximately halved (Figure 4 and Figure 7). However, the corresponding H_C_AB hybrid displays slightly superior binding to synaptosomes than H_C_B, which exactly reflects the biological activity of scBBAB [53]. In contrast, synaptosomal binding of H_C_BA is halved compared to H_C_A [53]; as is the SV2C binding of scAABA, but its GT1b binding is maintained (Figure 5 and Figure 7). Reduced SV2C binding of scAABA can be explained by the proximity of the SV2C binding site to the foreign H_CN_B, which might not support this interaction (Figure 1). Altogether, at least the H_CN_ domains of serotype A and B are largely exchangeable. Whether this relies on a specific structural feature in addition to the overall lectin-like fold present in both serotypes and whether the other known BoNT serotypes also comprise this feature needs to be investigated in future studies.

In contrast to swapping H_CN_A and H_CN_B, the substitution with H_CN_T decreased the biological activity of scAATA 5-fold and that of scBBTB 160-fold compared to the respective BoNT wild-types (Figure 3B). The synaptosomal binding of the corresponding H_C_TA is clearly reduced compared to H_C_A and H_C_T [54], but the SV2C binding of scAATA is only halved, while its GT1b binding is maintained (Figure 5 and Figure 7), a situation already seen with scAABA. The severe reduction in potency of scBBTB can neither be explained by its unaltered Syt-II interaction and the 50% reduced binding to GT1b (Figure 4 and Figure 7) nor by synaptosomal binding of the corresponding H_C_TB hybrid, which is close to that of H_C_B [54]. One speculation would be that scAATA and scBBTB are properly folded molecules and specifically bind to their neuronal receptors, but are then preferably sorted into neutral vesicles like TeNT, thereby failing to block acetylcholine release at the neuromuscular junction. For scAATA, this hypothesis was tested by intoxicating primary cultures of cortical neurons from the CNS [54]. However, activity of AATA was about 10-fold lower than of BoNT/A wild-type, which rejects this hypothesis. In conclusion, the H_CN_ of BoNTs are more compatible to each other than to H_CN_ of TeNT, probably due to their greater AA identity in the H_CN_ domain as well as due to the localization of the main receptor binding sites within H_CC_.

Additionally, the H_CN_ domain of BoNT/A was substituted with non-clostridial polypeptides of similar size like the 26 kDa GFP, the 21 kDa DHFR, or the peptidic linker L12. Whereas scAAL12A behaves like the BoNT/A deletion mutant scAA-A, the substitution of the H_CN_ domain by the rigid structure of GFP or the flexible structure of DHFR decreased biological activity of those hybrids below that of the deletion mutant scAA-A and abolished any binding to SV2C. Hence, proteins of similar size but different folding that could act as a rigid spacer do not harmonize with the surrounding translocation domain H_N_ and the H_CC_ domain, suggesting that H_CN_ is more than just an extended spacer domain. The presence of H_CN_ is vital for CNTs to exert their neurotoxicity. It might either orientate the H_CC_ domain for proper receptor interaction or subsequent to receptor binding positions the H_N_ domain in a pH dependent mechanism for membrane insertion as suggested by Montal [59]. Along that line, the helix connecting H_N_ and H_CN_ of BoNT/A was identified in playing an important role in pH-dependent orientation of the H_C_ fragment, which is important for formation of the 290 kDa M-PTC as well as for neuronal action [60]. Besides H_N_, the H_CN_ mediates most of the interactions between BoNT/A and E and their respective non-toxic non-hemagglutinins (NTNHA) as observed in the crystal structures of the M-PTC/A and E, respectively [60,61]. For TeNT, Masuyer et al. 2017 reported a pH-dependent movement of TeNT H_C_, which is stabilized by novel hydrophobic interactions between a strand of H_CN_ and a loop within LC further corroborating a role of H_CN_ in domain orientation [10].

Although structurally similar, the H_CN_ domain of TeNT cannot equally substitute those of BoNT and vice versa, leaving the possibility that H_CN_T plays a different role in the intoxication mechanism of TeNT like the sorting for retrograde axonal transport.

## 4. Conclusions

A main function of the H_CN_ domain besides contributing to N-glycan binding of SV2 in BoNT might be the correct positioning of the H_CC_ domain relative to the remaining LH_N_ of BoNT to provide access of the synaptic protein receptor to its binding pocket as well as to facilitate a subsequent pH-dependent insertion of H_N_ into the vesicle membrane. Proteins of similar size but different folding, which could act as a rigid spacer, cannot adopt that role.

## 5. Materials and Methods

### 5.1. Plasmid Construction

Expression plasmids encoding the full-length BoNT/A, BoNT/B, and TeNT (pBoNTAS, pBoNTBS, pAR1) have been described previously [25,33] and were used, for example, as template DNA for amplification of gene fragments encoding H_CC_, H_CN_, and H_C_. To easily swap the genes encoding the different H_CN_ and H_CC_ domains, a Nhe I site was inserted by PCR in pBoNTAS, pBoNTBS, and pAR1, causing the mutations N1093A in BoNT/A, Y1080A in BoNT/B, and I1111A/T1112S in TeNT. Analogously, a Sal I site was inserted by PCR in pBoNTAS, pBoNTBS, and pAR1 causing the mutations K871V/N872D in BoNT/A, S858V/E859D in BoNT/B, and I880D in TeNT to switch genes encoding the H_CN_ fragments of BoNT/A, B, and TeNT or to replace H_CN_A by GFP, DHFR, or L12 (=[Gly-Ser]6, GlyGlySerGlySerSerGlySerSerGlyGlySer) -linker, respectively. Accordingly, scAA-A lacks residues K871-N1093, scBB-B lacks S858-Y1080, and scTT-T lacks I880-I1111, respectively, while scAA—is devoid of residues 872–1296. In addition, the loop region connecting LC and HC of BoNT/A was substituted with a thrombin recognition sequence (LVPRGS) to allow specific activation of BoNT/A based constructs only by thrombin, as trypsin causes degradation of BoNT/A. The resulting plasmids pAtAAAS, pBBBBS, and pTTTTS (each capital letter encodes one of the following protein domain: LC, H_N_, H_CN_, H_CC_, t encodes the thrombin site; Figure 2A) quasi encoded BoNT/A, B, and TeNT wild-type [53,54]. Nucleotide sequences of all newly generated constructs were verified by DNA sequencing (GATC Biotech, Konstanz, Germany).

### 5.2. Expression and Purification of Recombinant Proteins

Recombinant full-length neurotoxins were expressed under biosafety level 2 containment (project number GAA A/Z 40654/3/123) in the *E. coli* strain M15[pREP4] (Qiagen, Hilden, Germany), following 16 h of induction at 22 °C. The clear lysates obtained from *E. coli* containing the single chain neurotoxins were ultracentifuged for 30 min at 35,000× *g* before purification on Streptactin-Superflow (IBA GmbH, Göttingen, Germany) according to the manufacturer’s instructions and kept in 100 mM Tris-HCl, pH 8.0. scH6tBoNTA and H_CC_A were purified as described earlier [15,39]. All recombinant proteins were shock frozen in liquid nitrogen and kept at −70 °C. After thawing of naïve aliquots, the purified CNT was centrifuged for 10 min at 20,000× *g* before use in an experiment to avoid protein aggregates. For circular dichroism (CD) analysis desired volume of protein was dialysed against PBS pH 7.4.

GST fusion proteins (GST-rSyt-II 1–61, GST-rSV2C 454–579) were purified as described earlier [39,43]. GST fusion proteins eluted by glutathione were dialyzed against PBS, pH 7.4, two times with, and two times without β-mercaptoethanol.

gH6thSV2CLDSFc and gSFc were purified from cell culture supernatants of transiently transfected HEK293T cells [47]. Briefly, one day before transfection, 18 × 10^6^ cells were seeded in 300 cm² culture flasks using growth medium (DMEM (Sigma-Aldrich Chemie GmbH, Taufkirchen Germany, D5671) supplemented with 10% FCS (PAN Biotech, P30-1502), 10 U/mL penicillin, 100 µg/mL streptomycin, and 2 mM L-glutamine). Transfection was performed by exchanging the growth medium of the culture and incubating 32 µg pIg+H6thSV2CLDS and 92 µL branched polyethylenimine (1 mg/mL, Sigma, #408727) in 4 mL DMEM without serum. Following 20 min of incubation at room temperature, the mixture was added to the culture. Transfection efficiency was estimated by replacing one-tenth of the amount of the DNA with the plasmid pEGFP-N2 (BD Biosciences Clontech, Stockholm, Sweden) and visual inspection of the green fluorescence of the cells one day after transfection. Following this protocol, the transfection efficiency was typically approximately 60%. Cell culture supernatants were collected two, four, and six days after transfection. gH6thSV2CLDSFc was consecutively affinity purified on Talon matrix (Clontech Laboratories, Inc., Mountain View, CA, USA) and Streptactin-Superflow (IBA GmbH, Göttingen, Germany) columns according to the manufacturers’ instructions and eluted using 100 mM Tris–HCl (pH 8.0), 150 mM NaCl and 10 mM Desthiobiotin.

Protein concentrations were determined subsequent to SDS-PAGE and Coomassie blue staining by using a LAS-3000 imaging system (Fuji Photo Film GmbH, Düsseldorf, Germany), the AIDA 3.51 software (Raytest, Straubenhardt, Germany), and BSA (100–1600 ng) as reference protein.

### 5.3. Potency of CNT Deletion Mutants and Hybrids at MPN Hemidiaphragm Assay

Potency of CNT deletion mutants and hybrids was determined employing the MPN assay [52,62]. In brief, the left phrenic nerve hemidiaphragm was excised from euthanized female mice of strain RjHan:NMRI (18–25 g, Janvier Labs, Genest Saint Isle, France), placed in an organ bath, and equilibrated for 15 min in 4 mL of Earle’s Balanced Salt Solution, pH 7.4, gassed with 95% O_2_ and 5% CO_2_. The phrenic nerve was continuously electro-stimulated (frequency 1 Hz, 0.1 ms pulse duration, 25 mA current). Isometric contractions (resting tension ~10 mN) were recorded with a force transducer (Scaime, Annemasse, France) and the software VitroDat (Föhr Medical Instruments GmbH (FMI), Seeheim, Germany). Toxin was applied in 4 mL of Earle’s Balanced Salt Solution supplemented with 0.1% BSA in concentrations to allow 50% decay of the contraction amplitude within 50 to 150 min. The times required to decrease the amplitude by 50% (paralysis time t_½_ ≤ 180 min) for three CNT concentrations were used to construct the calibration curves to which a power function was fitted (y (scAAAA; 30/100/270 pM) = 710.95x^−0.443^, *R*^2^ = 0.9925) (y (scBBBB; 90/270/890 pM) = 798.4x^−0.4^, *R*^2^ = 0.8952) (y (scTTTT; 2.0/6.5/20 nM) = 7586.8x^−0.492^, *R*^2^ = 0.9999). Each power function was used to convert the paralysis times t_½_ determined for deletion mutants and hybrids into the corresponding wild-type concentrations and expressed as relative biological activity of scAAAA wild-type.

### 5.4. GST-Pull-Down Assay

The GST-pull-down assays were similarly performed as previously described [15,27].

Briefly, GST and GST fusion proteins (150 pmol each) were immobilized to 10 µL glutathione-sepharose-4B matrix (Qiagen, Hilden, Germany) and subsequently incubated for 2 h at 4 °C with 250 nM CNTs in binding buffer (Tris-HCl pH 8, 150 mM NaCl, 0.5% Triton X-100). Beads were collected by centrifugation and washed two times each with the corresponding binding buffer. Washed pellet fractions were incubated at 37 °C for 20 min in SDS sample buffer and analyzed by 10% SDS-PAGE. Protein bands were detected by Coomassie blue staining and subsequently quantified by 2D densitometry using the software TINA (version 2.09f, Raytest, Straubenhardt, Germany). Coomassie blue staining of BoNT/A and B vs. BSA in SDS-PAGE with subsequent densitometry was compared to A280 photometry and found very consistent results, indicating that also the BoNT hybrids used here similarly stain to each other. Unspecific binding of ligand to immobilized GST was subtracted from the BoNT specific binding signal. The value “mol% binding” is the ratio of band intensity of the respective GST fusion protein as immobilized bait and the bound BoNT as ligand/prey corrected by the molecular weight ratio of BoNT vs. GST fusion protein.

Binding of wild-type and mutant ghSV2CLD Fc to scH6tBoNTA wild-type and the deletion mutant scAA-A, respectively, was described in [47]. Briefly, the assay was carried out in binding buffer (20 mM Tris-HCl, pH 7.4, 150 mM NaCl and 0.5% Triton X-100). A total of 10 µL protein G-sepharose beads (GE Healthcare) coated with 50 pmol of ghSV2CLD Fc was incubated with 50 pmol scH6tBoNTA or scAA-A, respectively, for 2–3 h in a total volume of 200 µL. Following incubation, beads were collected by centrifugation at 4500× *g* and washed three times using binding buffer. Washed pellet fractions were incubated in sodium dodecyl sulphate (SDS) sample buffer for 5 min at 99 °C and analyzed by SDS-PAGE. Proteins were visualized by means of Coomassie Blue staining.

### 5.5. Liposome Binding Assay

The Rhod-DHPE/GT1b-liposomes (41.98% Egg-PC (8.396 µM), 41.98% Cholesterol (8.396 µM), and 9.88% RhodamineB-DHPE (1.976 µM), 6.17% GT1b (1.234 µM)) were kindly provided by O.G. Weingart (ETH Zurich, Switzerland) and prepared by hydration of a thin film of dried lipids. Egg-PC, cholesterol, RhodamineB-DHPE, and GT1b ganglioside receptor molecules were dissolved in chloroform and methanol (1:1) in a glass vial by gentle agitation. Subsequently, the solvent was evaporated by applying a vacuum to create a dry lipid film, which was reconstituted in 12.5 mM HEPES pH 7.4 buffer. Shaking at 4 °C for 60 min resulted in a turbid vesicle emulsion. Following lipid film hydration, unilamellar LUV with uniform size distribution were produced by repeated extrusion of the vesicle emulsion through nucleopore track-etched polycarbonate membranes of 400 nm, 200 nm, and finally 100 nm pore size, flanked by a double stack of polyethylene drain discs on each site (Whatman plc, Kent, UK) assembled in a Mini-Extruder (Avanti Polar Lipids Inc., Alabaster, AL, USA). Liposomes were stored until use in a N_2_ atmosphere at 4 °C in the dark. Dynamic light scattering measurement revealed a mean diameter in different liposome dilutions between 174.7 and 176.6 nm and a polydispersity of 0.112–0.146.

A microtiter plate (Greiner Bio One Microplate, Fluotrac 600, high binding; black) was coated with 500 nM CNT in 100 µL diluted in PBS and shaken on a plate shaker at 4 °C overnight. After two washing steps with 200 µL/well PBS, Rhod-DHPE/GT1b-liposomes were diluted 1:500 in PBS to a concentration of 100 nM, and 100 µL was added per well. The covered plate was incubated for 2 h at 22 °C and 300 rpm. Following two additional washing steps, 120 µL of lysis buffer (0.1% Triton X-100 diluted in ddH_2_O) was added to the wells. After an incubation of 15 min at 22 °C and 300 rpm, the fluorescence signal was read in a fluorescence plate reader (BioTek Synergy^TM^ 4) using an excitation filter of 530 nm and emission wavelength of 585 nm at 30 °C.

### 5.6. Circular Dichroism Analysis

Circular dichroism (CD) data was collected with a Jasco J-810 spectropolarimeter in a 1 mm path length cuvette with a concentration of 2 µM respective protein in PBS pH 7.4. Spectra were recorded at 22 °C from 195 to 250 nm with 100 nm/min, response of 1 s, standard sensitivity, bandwidth of 1 nm, and five accumulations. Spectra were analyzed, processed, and visualized using Spectra Manager II software (JASCO International Co. Ltd., Tokyo, Japan). Secondary structure estimation was conducted using the SSE module of Spectra Manager II applying Yang reference.

## Abbreviations

The following abbreviations are used in this manuscript:

AA	amino acid
BoNT	botulinum neurotoxin
CD	circular dichroism
CNTs	clostridial neurotoxins
Rhod-DHPE	sulforhodamine 1,2-dihexadecanoyl-SN-glycero-3-phosphoethanolLamine, triethylammonium salt
Egg-PC	egg phosphatidylcholine
GBS	ganglioside binding site
GST	glutathion-S-transferase
h	human
HC	heavy chain
H_C_	carboxyl-terminal half of HC; H_CN_A, H_CN_B, H_CN_T, H_CN_ domain of BoNT serotype A, B, and TeNT, respectively; H_CN_ and H_CC_: 25 kDa halves of H_C_
H_N_	amino-terminal half of HC
HSA	human serum albumin
LC	light chain
LD	luminal domain
MPN assay	mice phrenic nerve hemidiaphragm assay
MW	molecular weight
PS	polystyro
S	C-terminal Streptag
SNAP-25	synaptosomal associated protein of 25 kDa
SNARE	soluble *N*-ethylmaleimide-sensitive factor attachment protein receptor
SV	synaptic vesicle
rSV2A-C	rat synaptic vesicle glycoprotein 2 isoform A, B, or C
Syt	synaptotagmin
TMD	transmembrane domain
